# Breast Cancer Classification by Using Multi-Headed Convolutional Neural Network Modeling

**DOI:** 10.3390/healthcare10122367

**Published:** 2022-11-25

**Authors:** Refat Khan Pathan, Fahim Irfan Alam, Suraiya Yasmin, Zuhal Y. Hamd, Hanan Aljuaid, Mayeen Uddin Khandaker, Sian Lun Lau

**Affiliations:** 1 Department of Computing and Information Systems, School of Engineering and Technology, Sunway University, Bandar Sunway 47500, Selangor, Malaysia; 2 Department of Computer Science and Engineering, University of Chittagong, Chittagong 4331, Bangladesh; 3 Department of Computer and Information Science, Graduate School of Engineering, Tokyo University of Agriculture and Technology, Koganei Campus, Tokyo 184-0012, Japan; 4 Department of Radiological Sciences, College of Health and Rehabilitation Sciences, Princess Nourah Bint Abdulrahman University (PNU), P.O. Box 84428, Riyadh 11671, Saudi Arabia; 5 Computer Sciences Department, College of Computer and Information Sciences, Princess Nourah Bint Abdulrahman University (PNU), P.O. Box 84428, Riyadh 11671, Saudi Arabia; 6 Centre for Applied Physics and Radiation Technologies, School of Engineering and Technology, Sunway University, Bandar Sunway 47500, Selangor, Malaysia; 7 Department of General Educational Development, Faculty of Science and Information Technology, Daffodil International University, DIU Rd, Dhaka 1341, Bangladesh; 8 Department of Engineering, School of Engineering and Technology, Sunway University, Bandar Sunway 47500, Selangor, Malaysia

**Keywords:** breast cancer classification, multi-headed CNN, ultrasound image processing, medical image modeling

## Abstract

Breast cancer is one of the most widely recognized diseases after skin cancer. Though it can occur in all kinds of people, it is undeniably more common in women. Several analytical techniques, such as Breast MRI, X-ray, Thermography, Mammograms, Ultrasound, etc., are utilized to identify it. In this study, artificial intelligence was used to rapidly detect breast cancer by analyzing ultrasound images from the Breast Ultrasound Images Dataset (BUSI), which consists of three categories: Benign, Malignant, and Normal. The relevant dataset comprises grayscale and masked ultrasound images of diagnosed patients. Validation tests were accomplished for quantitative outcomes utilizing the exhibition measures for each procedure. The proposed framework is discovered to be effective, substantiating outcomes with only raw image evaluation giving a 78.97% test accuracy and masked image evaluation giving 81.02% test precision, which could decrease human errors in the determination cycle. Additionally, our described framework accomplishes higher accuracy after using multi-headed CNN with two processed datasets based on masked and original images, where the accuracy hopped up to 92.31% (±2) with a Mean Squared Error (MSE) loss of 0.05. This work primarily contributes to identifying the usefulness of multi-headed CNN when working with two different types of data inputs. Finally, a web interface has been made to make this model usable for non-technical personals.

## 1. Introduction

Breast cancer (BCa) is a disease that develops from breast tissue. The common signs of BCa may include a lump in the breast, a deformed shape of the breast, the appearance of dimpling on the breast skin, emission of fluid from the nipple, the appearance of an inverted nipple, and a pink or scaly patch of skin. The two common types of BCa are (a) invasive ductal carcinoma (IDC) and (b) ductal carcinoma in situ (DCIS). The DCIS occurrences are 20–53%, and this type is somewhat less hazardous than the IDC, which encompasses the whole breast tissue [1].

The ever-growing population of Bangladesh and the lack of appropriate information might prompt an expanded number of patients with malignancy, which is inevitable, and a populace-based study shows that the essential obstruction to the early identification of BCa in Bangladeshi women is an absence of comprehension of screening to identify the beginning phase of the disease [2]. The occurrence of breast cancer is reported to be 19.3 per 100,000 Bangladeshi women aged between 15 and 44 years. Most Bangladeshi women assume that mass in the breast is the main indication or symptom of breast cancer. In addition, most of them are not adequately aware of the danger of breast cancer [3]. In Bangladesh, the lower level of attention to BCa may be attributed to low proficiency, minimal broad communications openness, neediness, and women’s situation in the families [4]. In Asia, Pakistan has the highest rate of breast cancer. Occurrence and death rates for breast cancer increase with age. Around 90,000 cases are accounted for yearly, causing a death rate of 40,000 [5]. The average age at which cancer was discovered in Pakistani women was in their 40 s. In 2008, around 182,460 cases were detected, and 40,480 died, in the United States [6]. Since the reasons for breast cancer remain obscure, early detection can diminish the passing rate (40% or more) [7]. However, early detection should be as much as precise and reliable that ought to distinguish benign and malignant tumors. 

One of the main strategies for the early detection of breast cancer is mammography [8]. However, there are some limitations of mammography in breast cancer classification. Many unnecessary (65–85%) biopsy activities are required due to the low explicitness of mammography [9]. Furthermore, mammography is not viable for solid breasts. In addition, the ionizing radiation of mammography can pose unnecessary hazards to patients and radiologists. Such techniques are favorably utilized compared to other techniques such as radiography, magnetic resonance imaging, and thermography. Ultrasound (US) imaging shows a growing interest in breast cancer identification [10,11,12]. It has been reported that more than one out of four patients are using ultrasound images for this purpose [13]. Studies have demonstrated that US imaging may accurately distinguish the benign and malignant types [14,15]. The usage of ultra-sound can extend in everyday malignant growth locations by 17% [16] and decrease the number of unnecessary biopsies by 40%, saving as much as $1 billion per year in the United States [17]. However, ultrasonography is considerably more operator-dependent than mammography; perusing ultrasound images requires an all-around ready and experienced sonographer. Undoubtedly, even all-around pre-arranged experts might have a high eyewitness variety rate. A decent finding approach should convey a low false positive (FP) rate and a false negative (FN) rate. Therefore, computer-aided diagnosis (CAD) is needed to help the technicians detect and classify BCa. A couple of CAD approaches have been considered to overcome the effect of operator-dependent errors in US imaging and to increase diagnostic affectability and particularity. Meraj et al. [18] used a CAD-like model based on quantization assisted U-net with BUSI and Open Access Database of Raw Ultrasonic Signals (OASBUD) datasets for doing segmentation. Initially, they segmented the lesion with U-Net, and later used Independent Component Analysis (ICA) to extract the features from it. Finally, these were combined with deep automated features. 

Machine learning, a sub-field of AI, assumes a significant part in the classification of breast cancer. Several studies use machine learning techniques such as linear discriminant analysis (LDA), support vector machine (SVM), and artificial neural network (ANN) for the classification and development of the model or to re-train the current models and for better execution. Some machine learning-based algorithms, such as KNN, Naive Bayes and Random Forest, are used for BCa with the Wisconsin Diagnosis dataset [19]. However, they did not mention any details of feature extraction. In [20], Ojha et al. showed the difference between the classification and clustering process for many ML algorithms and found that classification shows a better result on detection compared with clustering and the highest accuracy they obtained was 81% for SVM. Jabeen K et al. [21] suggested the fusion of deep learning features with manually selected features and used the CSVM classifier which produced a 99.1% detection accuracy. A Dilated Semantic Segmentation Network (Di-CNN) [22] with a morphological erosion operation has been used to segment images of ultrasound breast lesions. They demonstrated a 24-layer CNN architecture with transfer learning to obtain the desired intensity on extracted features. The feature vectors from DenseNet201 and the 24-layer CNN were merged using parallel fusion utilizing 10-fold cross-validation on different vector combinations to categorize the nodules. When paired with the Support Vector Machine (SVM) classifier, CNN-activated feature vectors and DenseNet201-activated feature vectors had accuracy rates of 90.11 percent and 98.45 percent, respectively. Another comparison between deep learning and machine learning algorithm for breast cancer detection has been conducted by Mekha et al. [23], but their feature extraction method is not clear. Apart from ML, most CAD systems need many diagnostic images to build the models or rules, yet Chen et al. [24] proposed an original system requiring very few samples. A lot of past examinations have proposed utilizing AI as Machine Learning and Deep Learning for image detection and healthcare monitoring [25,26,27,28,29]. Many fancy, heavy CNN variants are used for BCa detection and classification. A pre-trained model has been used by tuning hyperparameters and using DenseNet and ResNet gives 100% accuracy with Adam and RMSprop optimizers [30]. However, the ResNet is too heavy and most probably overfitted the ultrasound images, and they were willing to use a multi-model with a different data source. Multi-task and multi-class-based CNN has been used with VGG16 [31] and DenseNet [32] to extract features. However, they extracted two output results based on separate fully connected layers. This approach creates a problem of identity mismatch which refers to a problem when some features are causing a misclassification. Most, probably they would cause the same misclassification on separate output layers. By using this method, they increased the accuracy from 82.9% to 83.3%, which is not much overall. Transfer learning-based models are also being used in this medical sector, specially VGG16 with fine-tuning by Hijab et al. [33]. As VGG16 is a very deep complex model architecture and designed for dense image processing, the ultrasound image processing is far simpler for this model, making it highly prone to overfitting. A StepNet architecture with neutrosophic processing and fuzzy c-means clustering in the final layers has been proposed for ultrasound image processing by Sivanandan et al. [34]. They combined the original image with processed images and augmented them for training, which increased the complexity and the clustered mask-like output they generated was given with the dataset which makes this process illogical. A similar concept to ours, a stacked ensemble CNN has been used for classification [35]. However, they only trained the original ultrasound images and avoided the masks for all three layers. An Inception-V3 architecture has been modified to adopt ultrasound imaging [36]. They also used the multi-view architecture to train the dataset with InceptionV3 backbone and pretrained weight as transfer learning and we know transfer learning is not so good in sensitive medical images. 

Along with CAD, Machine Learning, Deep Learning models, and many intelligent systems have been developed to evaluate the risk and early age detection of BCa. An expert system based on Mamdani fuzzy-login inference type has been proposed to analyze patients’ healthcare data and interpret those data to generate a cancer risk level [37]. Upon collecting the data from their software and creating the knowledge base, they used KNN and Bagged Trees classifiers to obtain the result. However, due to reliability issues in predicted values and not having enough knowledge base data, this system is still not in use in real-time decision-making. A hybrid intelligent model consisting of a self-organized map (SOM) and complex-values neural network (CVNN) has been proposed for reliable BCa detection with 95% disease detection [38]. They claim to be reliable, but the regeneration of their model is too complicated and depends on six types of data. Another intelligent fuzzy temporal rule is being introduced to assess the risk of BCa by using a questionnaire-based user interactive module [39]. They have used Wisconsin dataset for training with a separate feature selection method. The details information about the logic behind fuzzy rules is missing, and the explainability of these rules is complicated.

From the literature and current other works, we came to understand that the methods recently being used are computationally expensive, prone to overfit, transfer learning-based method, single input but multi-output based, or heavy backbone-based method. To overcome some of these issues, in this work, we have applied multi-headed CNN, which takes processed original image and ground truth masked image contour values to detect normal, benign, and malignant types of breast cancer. The main goal is to combine two datasets so that they could complement each other to solve the problem of wrong classifications. Moreover, the custom CNN is very lightweight, simple, and computationally easy to train without any need for a high-end GPU, and a web application system has been built so that general people can use this model without any prior technical knowledge. 

The remaining sections are divided as follows: Section 2 describes materials and methods including the dataset we used, the main working procedure with a subsection of masked image processing and raw image processing, Section 3 discusses our methods, and, finally, Section 4 concludes our work.

## 2. Materials and Methods

### 2.1. Dataset

**Figure 1:**
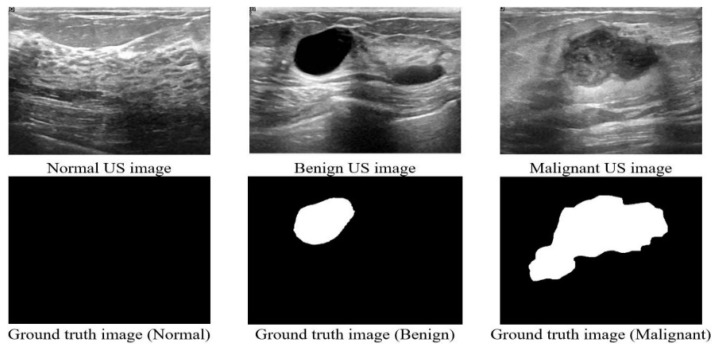
Sample images from the dataset.

We have analyzed the ultrasound images of breast cancer containing three classes (Benign, Malignant, and Normal). Multiple medical researchers used their own collected dataset [24,31]. Another popular dataset is Wisconsin Diagnostic which consists of 32 numeric features [40]. However, we wanted to use an image dataset, so we have used a publicly available ultrasound dataset which was released in 2018, the Breast ultrasound images dataset (BUSI) [41], collected from women between 25 and 75 years old, and the dataset details are shown in Table 1. A sample of the dataset is shown in Figure 1. This dataset has two types of images: raw images scanned by the LOGIQ E9 ultrasound system and masked ground truth images. We have used the masked images as a separate image base to combine the image and numeric data as multi-head CNN’s input layers. The total number of images is 830 and 75% of these images are used for training and 25% for testing.

### 2.2. Working Procedure

**Figure 2:**
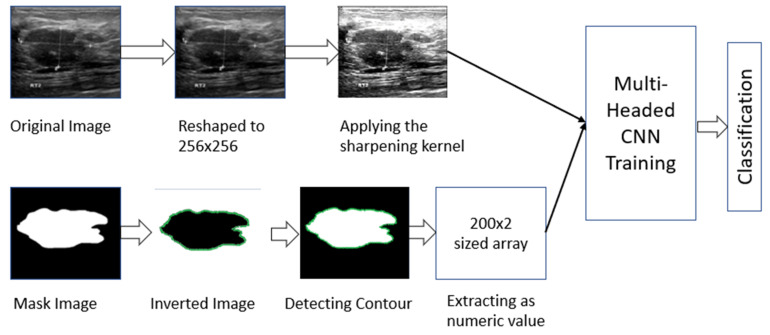
Working procedure of multi-headed concept.

The dataset contains two types of image data. As a result, it was necessary to process both types separately. For the masked images, we determined the contour area of the masked portion (details are described in Section 2.2.1). For the original image, we first performed some pre-processing (details are described in Section 2.2.2) and then used a CNN model to fit those images. Later, we used the individual model to combine and train the two types together to complement one another’s errors. The total working procedure is shown in Figure 2.

#### 2.2.1. Masked Image Processing

**Figure 3:**
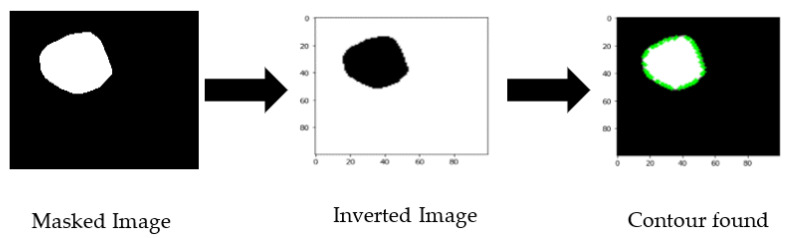
Masked image processing sequence.

The initial idea was to obtain the numeric polygon value for the covered mask area of the cancer portion for one training branch of CNN. To achieve this, we have processed masked images to obtain the border of the cancer part by exporting the contour area. First, we inverted the color and resized the image to 100 × 100 pixels, then giving the (255,255) threshold, the coordinates of the contour area were exported (shown in Figure 3). The exported contour values were not uniform, so we needed to add padding values to make all the extracted values the same length. We obtained the maximum length of contour points and reshaped them as numerical coordinate data into a 2D array of shapes (200, 2).

#### 2.2.2. Raw Image Processing

**Figure 4:**
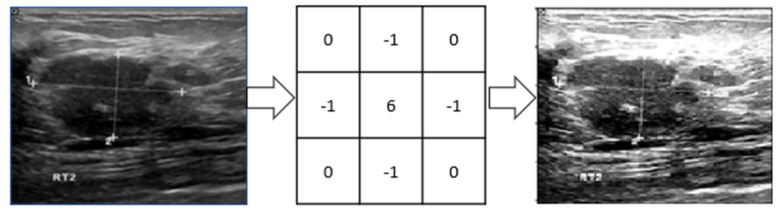
A 3 × 3 image sharpening kernel has been applied to sharpen images.

The original images are too large, which would increase the computational complexity. We have taken the original grayscale image to reduce the size and resized them to 256 × 256 pixels. After resizing, a 3 × 3 image sharpening kernel was used, as shown in Figure 4. The kernel reduces some image distortion. Then images’ pixel values were normalized between [0, 1] by dividing them by 255 as they are grayscale pixel values.

### 2.3. Modeling and Evaluating Datasets

#### 2.3.1. Evaluating Masked Images

**Figure 5:**
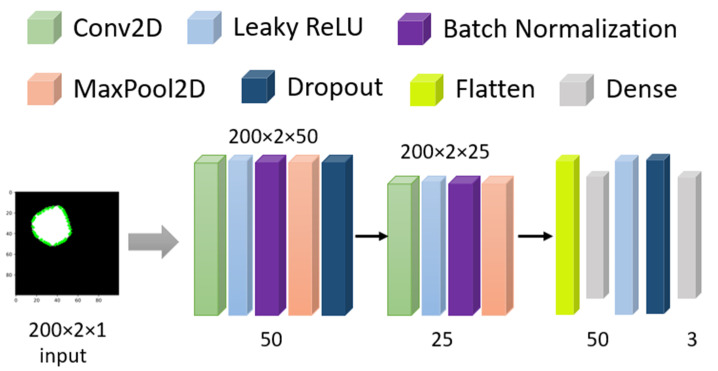
Model architecture for Masked images.

From Section 2.2.1, we have a 2D array of processed masked coordinates of shapes. Now, that dataset is evaluated through a Conv2D model (shown in Figure 5) with 100 epochs and a dynamic learning rate reduction method (monitoring validation accuracy with factor 0.5 and minimum learning rate of 0.00001) to avoid overfitting. For training, 75% of the data and for testing, 25% data has been used. The initial input size is 200 × 2 as the data format with 50 neurons and 0.1 as alpha in the LeakyRelu, followed by BatchNormalization, 2 × 2 MaxPooling with 1 Stride and 30% Dropout. In the second cell, the neuron decreased to 25, and alpha increased to 0.2 for LeakyRelu. Finally, with 100 Dense units in the flattened layer, the result has been extracted with a softmax function. This model has 512,128 trainable parameters.

**Figure 6:**
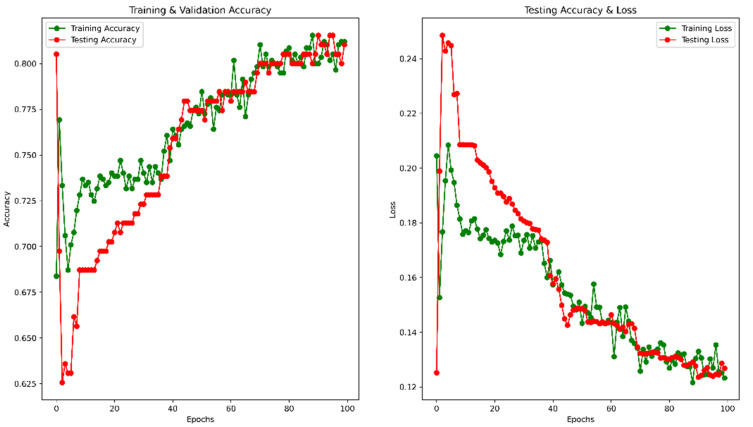
Accuracy and Loss vs. Epoch for masked image training.

**Figure 7:**
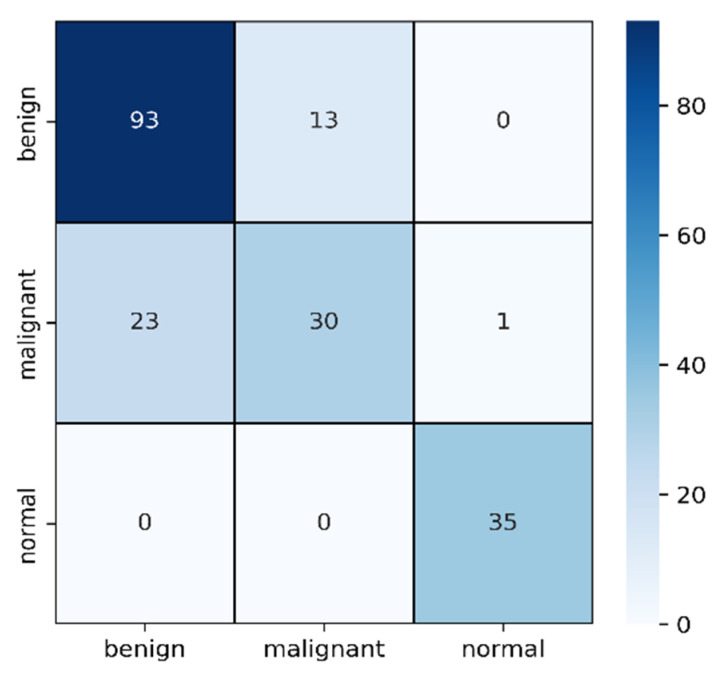
Confusion matrix for masked image evaluation on 25% of test data.

After 100 epochs, the validation accuracy was achieved at 82.91%. The accuracy and MSE loss vs. epoch graph are shown in Figure 6. The precision, recall, F1 score, and confusing matrix for the masked image processing model in classification are shown in Table 2 and Figure 7.

In Table 2, the F1 score of malignant is comparatively lower than the other two types though the sample number is medium. Among 54 samples, 23 have been detected as benign and the other 1 as normal. As the differences between malignant and benign from images are very difficult to detect where shapes are slightly differentiable, it is understandable why these two became confused. On the other hand, the 93 benign cases are successfully detected and confused only 13 cases with malignant ones.

#### 2.3.2. Evaluating Original Images

**Figure 8:**
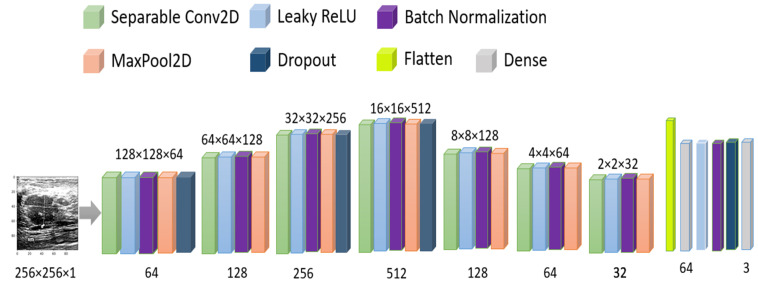
Model architecture for original image training.

As the number of images is comparatively low, we have used image augmentation as horizontal flip, 10° random rotation, 0.1 random zoom range, and 0.1 height and width shift. In the CNN model, we have used separable conv2D by 64, 128, 256, 512, 128, 64 neurons with stride = (2,2) and same padding, LeakyRelu with 0.1 alpha, batch normalization, max-pooling with pool size = (2,2), and 20% dropout with adam optimizer and MSE loss. detailed model architecture has been shown in Figure 8. The total number of parameters used is 6,575,576.

**Figure 9:**
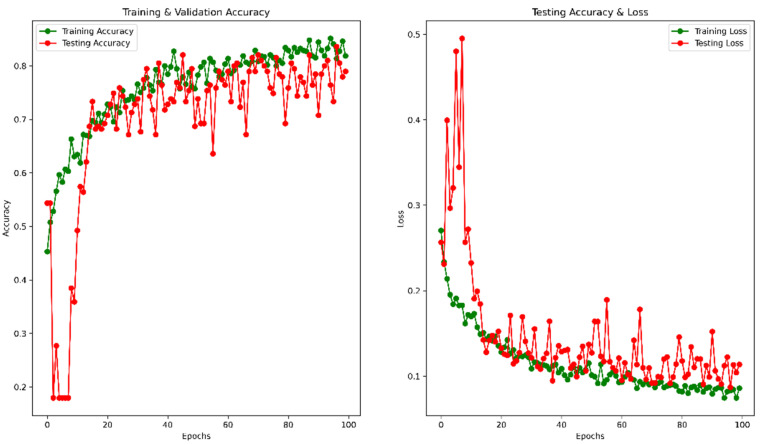
Accuracy and Loss vs. Epoch for original image in classification.

**Figure 10:**
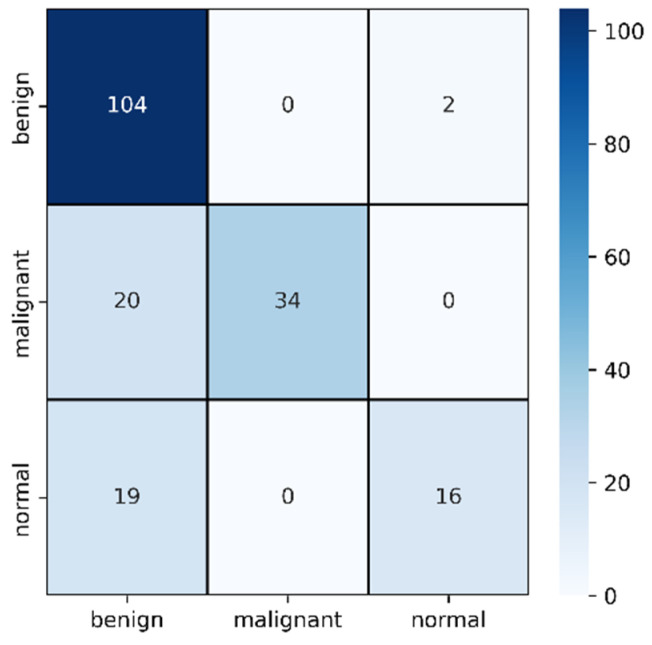
Confusion matrix for original image evaluation on 25% of test data.

To avoid overfitting, we used the dynamic learning rate reduction function “ReduceLROnPlateau” to monitor the validation accuracy, patient = 2, factor = 0.5, and minimum learning rate as 0.00001. After 100 epochs, the validation accuracy reached 78.97%, and the MSE was 0.11. The accuracy vs. epoch and loss vs. epoch graphs are shown in Figure 9. The precision, recall, F1 score, and confusing matrix for the original image processing model in classification have been shown in Table 3 and Figure 10.

In Table 3, the F1 score of malignant improved from the previous section to 77% and misclassification was reduced to only 20. However, this time, the model failed to identify normal cases and misclassified 19 cases as benign. However, the benign classification improved from mask images where only two images are identified as normal. In this case, the benign and malignant seems to be classified accurately but normal cases are creating the confusion.

#### 2.3.3. Validation of Multi-Headed CNN

**Figure 11:**
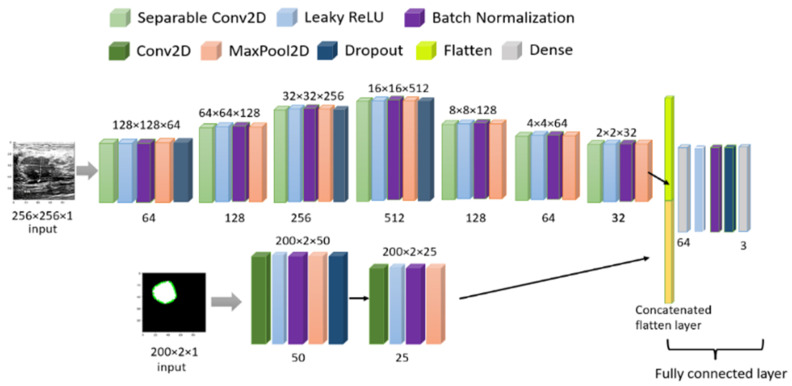
Model architecture for combined image training.

Finally, we have combined the original image and masked image inputs with a two-headed CNN model to train parallel models, giving one output series of three classes. The initial learning was the default TensorFlow value, but to avoid the overfitting problem, a callback method was used for a dynamic learning rate reduction. The batch size was 20, and the training-testing set was 75–25% with 100 epochs. The final CNN model is shown in Figure 11 and all the related parameters and layers are described in Table 4. We have used the Google Colab free version to train our model. The free version gives us 12 GB RAM and 15 GB GPU. Training of 100 epochs with the multi-headed model utilized 3.63 GB RAM and 4.99 GB GPU and each epoch took 6 s (220 ms/step) for 29 steps. After training, we saved the model in h5 format for use with the web interface, and the model weight size is only 4.64 MB.

**Figure 12:**
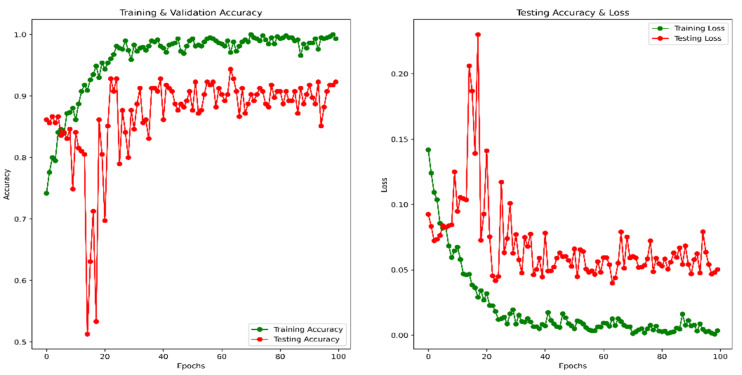
Accuracy and Loss vs. Epoch for a combined image in classification.

**Figure 13:**
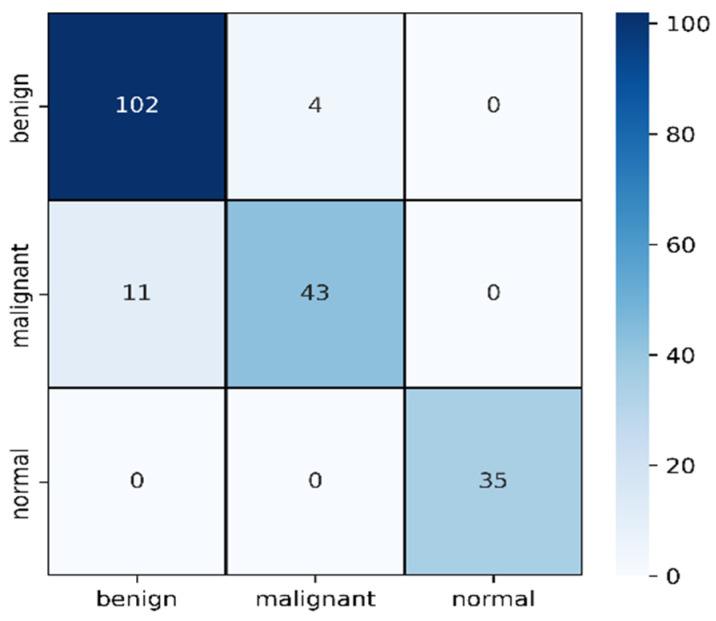
Confusion matrix for combined image evaluation on 25% of test data.

**Figure 14:**
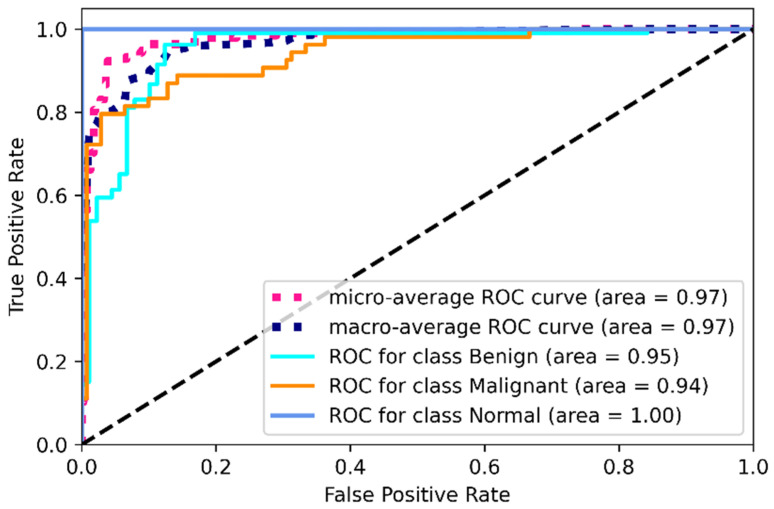
ROC for combined image evaluation (on training data).

Using the same image augmentation variables and after 100 epochs, the accuracy and loss vs. epoch have been shown in Figure 12. These graphs show a step-by-step improvement, eliminating the possibility of data overfitting. After around 80 epochs, the accuracy and loss values seem to be bouncing up and down, not maintaining a smooth flow, probably due to the lower epoch number. The precision, recall, F1 score, and confusing matrix for the mixed data processing model in classification have been shown in Table 5 and Figure 13. Finally, the ROC has been shown for this model evaluation in Figure 14.

From Table 5, we can clearly see the improvement in mask and raw image processing results. This time, the F1 score improved in all three categories, especially for the malignant category. This one is likely to be identified as benign on mask processing, but in this combined scenario, the detection rate was improved and only 11 cases were misclassified causing an F1 score of 85%. The benign improved from 84% average F1 score to 93%, misclassifying four cases. On this combined architecture, the normal cases are highly improved in detection with 0 misclassifications. From the ROC curves, the ROC values on training dataset for benign, malignant and normal are found to be 95%,94%, and 100%.

### 2.4. Web Application Interface

**Figure 15:**
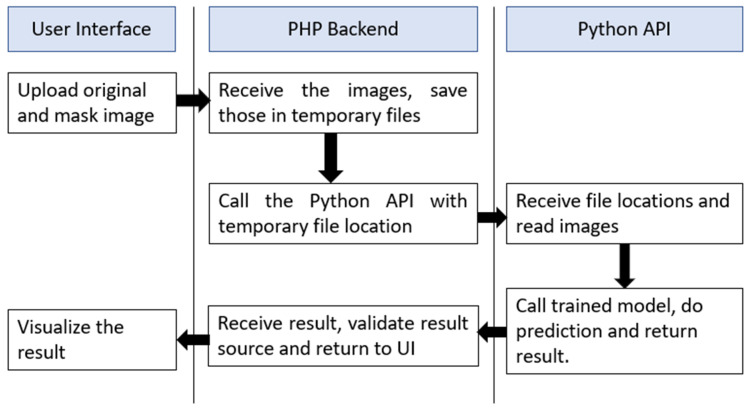
The software flowchart for the web interface of breast cancer detection.

To make this system available for non-technical persons, we have developed a web interface in localhost with PHP—v7.4 backend. The system takes two images as input, one is the original ultrasound raw image and another one is the mask of that image. These two images are processed and sent to a python—v3.8 code by AJAX for prediction. Python receives these two images, performs the processing and returns the processed pre-input image format and the predicted class in JSON format. Upon receiving the JSON output, AJAX shows the result images and classification results in real-time. The software flowchart is shown in Figure 15. After further development and proper hosting, this site will be published in a live website interface.

## 3. Discussing of Result

The CNN-based proposed breast cancer identification technique has achieved an optimal outcome. We have used ultrasound images of breast cancer containing three classes (Benign, Malignant, and Normal). Training and testing data were performed through three stages: (a) masked image processing having validation with 81.02% accuracy without creating overfitting, (b) original image processing with validation accuracy of 78.97%, and (c) multi-headed CNN with 92.31% validation accuracy. We combined the datasets for experimenting with our model with dynamic learning rate reduction and trained 75% of the data; the remaining 25% was used for testing. The Google Colab platform has been used for coding, and the Keras library was used for building the model. Some comparisons of methodology with findings have been shown in Table 6.

In the literature, we can see much higher accuracy [20,23,25,32,33] compared with our results, but most of them have reliability [35,36] issues or complex training issues [30,31]. The result we achieved is only after 100 epochs, if we run higher epochs, there is a high chance that this accuracy will improve. However, we wanted to keep the computational cost low with an acceptable better result. We have used the BUSI dataset because it has the mask we considered as a completely different type and converted to numeric data as this is not a segmentation task. In our method, it is possible to utilize the model for similar types of datasets that could be extracted into multiple parts. Though our final accuracy was lower than some of the existing literature, we showed that the use of multiple CNN channels could complement the single channel’s weights. The individual channel weight and combined weights are making a difference in accuracy by around 10%, as described above. Later we are planning to perform a segmentation task with the popular Mask R-CNN [42] on it as the ground truth is already extracted. Segmentation will solve our primary limitation of having mask value in the testing procedure, improving the model’s usability in real-life applications. Marking and extracting the contour area from the binary image mask gives some idea about how the model is detecting classes which refer to the explainability of the model. Still, there are some limitations to model explainability to be exact, which need further work, especially in medical images.

## 4. Conclusions

As breast cancer is now a specific disease in women, the early identification of these diseases should be dealt with more effectively in contrasting and genuine conditions. The average 10-year endurance rate for females with obtrusive breast cancer is 84%. On the off chance that the invasive cancer is found uniquely in the breast, the 5-year survival rate of women with breast cancer is almost 99%, and 62% of women with breast cancer are diagnosed with this stage. The fundamental objective of this study is to find how to utilize multi-set CNN in the simple and cost-effective detection of breast cancer by using ultrasound images. We have utilized breast cancer identification using two-channel input datasets with a multi-headed CNN, achieving 92.31% (±2) accuracy. The proposed model was discovered to effectively acquire the actual outcomes that may diminish human mistakes in the diagnosis process and reduce the cost of a cancer diagnosis. We also developed a web-based system to give a trial to real-time analysis, which will be made publicly available soon. Moreover, our study depends on essential primary data for more exactness of the outcomes identified with breast cancer identification. This work implies using multiple types of data to achieve one target, so that features can complement each other in case of a wrong decision in one channel. This kind of multi-headed network is logically more usable in the medical field, where different kinds of data are needed to be exactly sure about the condition of patients. As reliability is very important in medical decisions, our work would create a new way of generating reliable knowledge.

In the future, we would like to acquire image segmentation so that the neural network can precisely detect cancer and the portion that causes cancer. Recently, the Mask R-CNN is one of the most popular models for instance segmentation, especially on medical images.

## Figures and Tables

**Table 1:** Details of breast cancer dataset.

Type	Number of Images	Shape	Color	Format
Benign	487	500 × 500 Pixel	Grayscale	png
Malignant	210			
Normal	133			

**Table 2:** Precision, Recall, and F1 score for the masked image on 25% of test data.

	Precision	Recall	F1 Score	Support
Benign	0.80	0.88	0.84	106
Malignant	0.70	0.56	0.62	54
Normal	0.97	1.00	0.99	35
accuracy			0.81	195
macro avg	0.82	0.81	0.81	195
weighted avg	0.80	0.81	0.80	195

**Table 3:** Precision, Recall, and F1 score for the original image on 25% of test data.

	Precision	Recall	F1 Score	Support
Benign	0.73	0.98	0.84	106
Malignant	1.00	0.63	0.77	54
Normal	0.89	0.46	0.60	35
accuracy			0.79	195
macro avg	0.87	0.69	0.74	195
weighted avg	0.83	0.79	0.78	195

**Table 4:** Parameter details of used Multi-headed model.

Layer (Type)	Output Shape	Param	Connected to
“input_2 (InputLayer)”	[(None, 256, 256, 1)]	0	[]
“separable_conv2d (SeparableConv2D)”	(None, 256, 256, 64)	137	“[input_2[0][0]]”
“leaky_re_lu_5 (LeakyReLU)”	(None, 256, 256, 64)	0	“[separable_conv2d [0][0]]”
“batch_normalization_4 (BatchNormalization)”	(None, 256, 256, 64)	256	“[leaky_re_lu_5[0][0]]”
“max_pooling2d_5 (MaxPooling2D)”	(None, 128, 128, 64)	0	“[batch_normalization_4[0][0]]”
“dropout_3 (Dropout)”	(None, 128, 128, 64)	0	“[max_pooling2d_5[0][0]]”
“separable_conv2d_1 (SeparableConv2D)”	(None, 128, 128, 128)	8896	“[dropout_3[0][0]]”
“leaky_re_lu_6 (LeakyReLU)”	(None, 128, 128, 128)	0	“[separable_conv2d_1[0][0]]”
“batch_normalization_5 (BatchNormalization)”	(None, 128, 128, 128)	512	“[leaky_re_lu_6[0][0]]”
“input_1 (InputLayer)”	[(None, 200, 2, 1)]	0	[]
“max_pooling2d_6 (MaxPooling2D)”	(None, 64, 64, 128)	0	“[batch_normalization_5[0][0]]”
“conv2d_2 (Conv2D)”	(None, 200, 2, 50)	500	“[input_1[0][0]]”
“separable_conv2d_5 (SeparableConv2D)”	(None, 64, 64, 64)	9408	“[max_pooling2d_6[0][0]]”
“leaky_re_lu_3 (LeakyReLU)”	(None, 200, 2, 50)	0	“[conv2d_2[0][0]]”
“leaky_re_lu_10 (LeakyReLU)”	(None, 64, 64, 64)	0	“[separable_conv2d_5[0][0]]”
“batch_normalization_2 (BatchNormalization)”	(None, 200, 2, 50)	200	“[leaky_re_lu_3[0][0]]”
“batch_normalization_9 (BatchNormalization)”	(None, 64, 64, 64)	256	“[leaky_re_lu_10[0][0]]”
“max_pooling2d_3 (MaxPooling2D)”	(None, 200, 2, 50)	0	“[batch_normalization_2[0][0]]”
“max_pooling2d_10 (MaxPooling2D)”	(None, 32, 32, 64)	0	“[batch_normalization_9[0][0]]”
“dropout_2 (Dropout)”	(None, 200, 2, 50)	0	“[max_pooling2d_3[0][0]]”
“separable_conv2d_6 (SeparableConv2D)”	(None, 32, 32, 32)	2656	“[max_pooling2d_10[0][0]]”
“conv2d_3 (Conv2D)”	(None, 200, 2, 25)	11275	“[dropout_2[0][0]]”
“leaky_re_lu_11 (LeakyReLU)”	(None, 32, 32, 32)	0	“[separable_conv2d_6[0][0]]”
“leaky_re_lu_4 (LeakyReLU)”	(None, 200, 2, 25)	0	“[conv2d_3[0][0]]”
“batch_normalization_10 (BatchNormalization)”	(None, 32, 32, 32)	128	“[leaky_re_lu_11[0][0]]”
“batch_normalization_3 (BatchNormalization)”	(None, 200, 2, 25)	100	“[leaky_re_lu_4[0][0]]”
“max_pooling2d_11 (MaxPooling2D)”	(None, 16, 16, 32)	0	“[batch_normalization_10[0][0]]”
“max_pooling2d_4 (MaxPooling2D)”	(None, 200, 2, 25)	0	“[batch_normalization_3[0][0]]”
“dropout_6 (Dropout)”	(None, 16, 16, 32)	0	“[max_pooling2d_11[0][0]]”
“flatten_1 (Flatten)”	(None, 10000)	0	“[max_pooling2d_4[0][0]]”
“flatten_2 (Flatten)”	(None, 8192)	0	“[dropout_6[0][0]]”
“concatenate (Concatenate)”	(None, 18192)	0	“[flatten_1[0][0],flatten_2[0][0]]”
“dense_2 (Dense)”	(None, 64)	1164352	“[concatenate[0][0]]”
“leaky_re_lu_12 (LeakyReLU)”	(None, 64)	0	“[dense_2[0][0]]”
“batch_normalization_11 (BatchNormalization)”	(None, 64)	256	“[leaky_re_lu_12[0][0]]”
“dropout_7 (Dropout)”	(None, 64)	0	“[batch_normalization_11[0][0]]”
“dense_3 (Dense)”	(None, 3)	195	“[dropout_7[0][0]]”
Total params: 1,199,127 Trainable params: 1,198,273 Non-trainable params: 854			

**Table 5:** Precision, Recall, and F1 score for the combined image on 25% of test data.

	Precision	Recall	F1 Score	Support
Benign	0.90	0.96	0.93	106
Malignant	0.91	0.80	0.85	54
Normal	1.00	1.00	1.00	35
accuracy			0.92	195
macro avg	0.94	0.92	0.93	195
weighted avg	0.92	0.92	0.92	195

**Table 6:** Comparison of existing methods with their findings and datasets.

References	Method	Datasets	Findings
[20]	Random Forest, KNN and Naïve Bayes.	Wisconsin Breast Cancer dataset from UCI Repository	KNN was an excellent classifier in terms of accuracy of 94%.
[19]	Data Mining techniques	Wisconsin breast cancer dataset.	Detect hidden cancer-associated for classification SVM accuracy 81%.
[23]	Machine learning techniques	Mammogram images.	It achieved 96% accuracy by using DNN
[32]	Deep learning	Wisconsin breast cancer dataset.	The author compared machine learning techniques and deep learning. It achieved 96.99% accuracy with DL.
[24]	CAD system	Custom ultrasound images	Accuracy with 10 samples and the bootstrap method is 87.07%
[25]	CAD system	Mammogram images.	Random forest gives the highest accuracy of 97.51%
[31]	Multi-task CNN	Custom ultrasound images	With a baseline of 83.3%
[32]	Multi-Class CNN	BreaKHis dataset	Image level accuracy 95.4%
[33]	VGG16 and transfer learning	BUSI	Fined tuned model accuracy is 97%
**Proposed Method**	Multi-headed CNN	BUSI	Multiple types of datasets could generate more significant results than solo datasets. Accuracy is 92.31% (±2)

## Data Availability

All data are available in the manuscript.
